# Rare and Common Variants in *GALNT3* May Affect Bone Mass Independently of Phosphate Metabolism

**DOI:** 10.1002/jbmr.4795

**Published:** 2023-03-13

**Authors:** Neelam Hassan, Celia L. Gregson, Haotian Tang, Marc van der Kamp, Paul Leo, Aideen M. McInerney‐Leo, Jie Zheng, Maria Luisa Brandi, Jonathan C. Y. Tang, William Fraser, Michael D. Stone, Elin Grundberg, Anglo‐Australasian Genetics Consortium, Matthew A. Brown, Emma L. Duncan, Jonathan H. Tobias

**Affiliations:** ^1^ Musculoskeletal Research Unit, Translational Health Sciences, Bristol Medical School University of Bristol Bristol UK; ^2^ MRC Integrated Epidemiology Unit, Population Health Sciences, Bristol Medical School University of Bristol Bristol UK; ^3^ School of Biochemistry University of Bristol Bristol UK; ^4^ Faculty of Health, Translational Genomics Group, Institute of Health and Biomedical Innovation Queensland University of Technology Brisbane Queensland Australia; ^5^ The Faculty of Medicine, Frazer Institute The University of Queensland Woolloongabba Queensland Australia; ^6^ Department of Endocrine and Metabolic Diseases, Shanghai Institute of Endocrine and Metabolic Diseases, Ruijin Hospital Shanghai Jiao Tong University School of Medicine Shanghai China; ^7^ Shanghai National Clinical Research Center for metabolic Diseases, Key Laboratory for Endocrine and Metabolic Diseases of the National Health Commission of the PR China Shanghai Jiao Tong University School of Medicine Shanghai China; ^8^ Shanghai Key Laboratory for Endocrine Tumor, State Key Laboratory of Medical Genomics, Ruijin Hospital Shanghai Jiao Tong University School of Medicine Shanghai China; ^9^ FIRMO Foundation Florence Italy; ^10^ Norwich Medical School University of East Anglia Norwich UK; ^11^ Clinical Biochemistry, Departments of Laboratory Medicine Norfolk and Norwich University Hospital NHS Foundation Trust Norwich UK; ^12^ Department of Diabetes, Endocrinology and Clinical Biochemistry Norfolk and Norwich University Hospital NHS Foundation Trust Norwich UK; ^13^ University Hospital Llandough Cardiff & Vale University Health Board Cardiff UK; ^14^ Genomic Medicine Center Children's Mercy Kansas City Kansas City Missouri USA; ^15^ Genomics England London UK; ^16^ Department of Twin Research and Genetic Epidemiology, School of Life Course & Population Sciences, Faculty of Life Sciences and Medicine King's College London London UK

**Keywords:** HIGH BONE MASS, EXOME SEQUENCING, MONOGENIC, GALNT3, PHOSPHATE

## Abstract

Anabolic treatment options for osteoporosis remain limited. One approach to discovering novel anabolic drug targets is to identify genetic causes of extreme high bone mass (HBM). We investigated a pedigree with unexplained HBM within the UK HBM study, a national cohort of probands with HBM and their relatives. Whole exome sequencing (WES) in a family with HBM identified a rare heterozygous missense variant (NM_004482.4:c.1657C > T, p.Arg553Trp) in *GALNT3*, segregating appropriately. Interrogation of data from the UK HBM study and the Anglo‐Australasian Osteoporosis Genetics Consortium (AOGC) revealed an unrelated individual with HBM with another rare heterozygous variant (NM_004482.4:c.831 T > A, p.Asp277Glu) within the same gene. In silico protein modeling predicted that p.Arg553Trp would disrupt salt‐bridge interactions, causing instability of GALNT3, and that p.Asp277Glu would disrupt manganese binding and consequently GALNT3 catalytic function. Bi‐allelic loss‐of‐function *GALNT3* mutations alter FGF23 metabolism, resulting in hyperphosphatemia and causing familial tumoral calcinosis (FTC). However, bone mineral density (BMD) in FTC cases, when reported, has been either normal or low. Common variants in the *GALNT3* locus show genome‐wide significant associations with lumbar, femoral neck, and total body BMD. However, no significant associations with BMD are observed at loci coding for FGF23, its receptor FGFR1, or coreceptor klotho. Mendelian randomization analysis, using expression quantitative trait loci (eQTL) data from primary human osteoblasts and genome‐wide association studies data from UK Biobank, suggested increased expression of *GALNT3* reduces total body, lumbar spine, and femoral neck BMD but has no effect on phosphate concentrations. In conclusion, rare heterozygous loss‐of‐function variants in *GALNT3* may cause HBM without altering phosphate concentration. These findings suggest that GALNT3 may affect BMD through pathways other than FGF23 regulation, the identification of which may yield novel anabolic drug targets for osteoporosis. © 2023 The Authors. *Journal of Bone and Mineral Research* published by Wiley Periodicals LLC on behalf of American Society for Bone and Mineral Research (ASBMR).

## Introduction

Osteoporotic fractures affect one in three women and one in five men over the age of 50 worldwide, causing substantial morbidity and mortality, with annual healthcare costs in the USA alone of over $20 billion.^(^ ^1^ ^)^ Osteoporotic fractures are the leading cause of hospitalization in women over the age of 45 in the UK, occupying more bed‐days than any other condition including breast cancer, diabetes, and cardiovascular disease.^(^ ^2^ ^)^ Antiresorptive drugs, such as bisphosphonates or denosumab, currently form the mainstay of osteoporosis treatment. Long‐term antiresorptive use is limited by concern regarding rare side effects, including atypical femoral fractures and osteonecrosis of the jaw. In contrast, anabolic treatments actively stimulate new bone formation. Until recently, parathyroid hormone (PTH) derivatives and analogs such as teriparatide (PTH^1‐34^) and abaloparatide (PTH‐related protein analog) were the only anabolic treatment options available but the requirement for daily subcutaneous injections and, in many countries, restrictions on prescribing limit their use.^(^ ^3^ ^)^ Romosozumab was recently approved as an anabolic treatment in the USA and Europe, with once‐monthly subcutaneous administration, stimulating bone formation while also reducing bone resorption.^(^ ^4^ ^)^ Despite clinical efficacy, concerns regarding cardiovascular side effects restricted approval by the European Medicines Agency to selected women with severe osteoporosis. Thus, there remains a need for safe, and preferably orally administered, anabolic treatments for osteoporosis.

Romosozumab is an excellent example of where data from human genetic studies can be used to identify novel drug targets. This agent, which consists of an anti‐sclerostin antibody, was developed after the discovery that mutations in and around *SOST*,^(^ ^5^, ^6^ ^)^ the gene encoding sclerostin, underlie the rare diseases sclerosteosis^(^ ^5^ ^)^ and van Buchem's disease^(^ ^7^ ^)^; common variants at this locus are also associated with bone mineral density (BMD) (reported in multiple genome‐wide association studies [GWASs]).^(^ ^8^ ^)^ Thus, a highly successful approach to anabolic drug discovery is to identify genetic causes underlying skeletal dysplasias associated with high bone mass (HBM), specifically HBM resulting from excessive osteoblastic bone formation. Both sclerosteosis and van Buchem's disease are associated with pathological features arising from bone overgrowth, such as nerve compression.^(^ ^9^ ^)^ In contrast, several other monogenic disorders have been identified where HBM due to excessive osteoblast activity is an incidental finding, caused by mutations in other Wnt signaling proteins, namely LRP5 and LRP6, usually without sinister consequences of bony overgrowth.^(^ ^10^ ^)^

The UK HBM study was established to identify new monogenic causes for HBM.^(^ ^11^ ^)^ Pathological variants in known HBM genes (*SOST*/*LRP5*) were identified in a small minority, leaving most with unexplained HBM.^(^ ^12^ ^)^ Although enrichment of common variant “high‐BMD” alleles in known BMD‐associated loci was evident,^(^ ^13^ ^)^ it is likely that further monogenic causes exist that are yet to be discovered. To examine this question, we undertook whole exome sequencing (WES) of our HBM population and analyzed the data using a bespoke pipeline developed to identify underlying monogenic variants in individuals and/or families with phenotypes of interest.^(^ ^14^ ^)^ Using this approach, we recently identified a rare missense pathogenic variant in *SMAD9*, segregating with HBM in an autosomal dominant pattern within a UK HBM pedigree.^(^ ^15^ ^)^ The same variant was identified in two additional unrelated individuals with HBM sequenced as part of the Anglo‐Australian Osteoporosis Genetics Consortium (AOGC).^(^ ^16^ ^)^ Moreover, common variants at the same locus are associated with BMD in the general population.^(^ ^15^ ^)^ These findings led to the identification of the SMAD‐dependent BMP signaling pathway as a potential anabolic target for osteoporosis treatment.

Both *SOST* and *SMAD9* gene discoveries highlight the value of investigating rare monogenic HBM in identifying novel anabolic drug targets. We sought to identify additional novel monogenic causes of HBM by analyzing WES data from further kindreds in our UK HBM study.

## Methods

### The UK HBM study

The HBM study is a UK‐based multicenter observational study of adults with unexplained HBM, identified incidentally on routine clinical dual‐energy X‐ray absorptiometry (DXA) scanning. Briefly, DXA databases containing 335,115 DXA scans across 13 UK centers were searched; all scans explained by artifact or known causes of high BMD were excluded, principally degenerative disease/osteoarthritis (OA). Unexplained HBM was defined as (1) first lumbar vertebra (L1) *Z*‐score of ≥+3.2 plus total hip (TH) *Z*‐score of ≥+1.2 and/or (2) TH *Z*‐score ≥+3.2 plus L1 *Z*‐score of ≥+1.2. Full details of DXA database screening and participant recruitment were previously reported together with the clinical, biochemical, and imaging assessments performed.^(^ ^11^, ^15^ ^)^ Baseline recruitment took place between 2005 and 2010 and a follow‐up study was performed between 2016 and 2018.^(^ ^17^ ^)^ The study recruited 337 individuals with unexplained HBM (240 probands and 97 affected relatives).

### Anglo‐Australasian Osteoporosis Genetics Consortium (AOGC) HBM and LBM cases

The AOGC extreme truncate population comprises 1128 Australian, 74 New Zealand, and 753 British unrelated women of white Caucasian ancestry, at age 55 to 85 years, ≥5 years postmenopausal, with either HBM (TH BMD *Z*‐scores +1.5 to +4.0, *n* = 1055) or low bone mass (LBM) (*Z*‐scores −4.0 to −1.5, *n* = 900), with no known secondary cause for altered BMD in either group.^(^ ^16^ ^)^

### Whole exome sequencing and analysis

HBM study probands (excluding individuals with pathogenic variants in known HBM genes) underwent WES. Following review of each HBM pedigree, family members were also selected who were related to the index case and had a phenotype and DNA collected. Where the pedigree spanned three generations, the most distant relatives were sequenced. Additionally, WES was performed in 126 HBM and 493 LBM individuals from AOGC, as well as 240 from the HBM study. Library creation, sequencing, base calling, sequence alignment, and variant calling were performed as previously described.^(^ ^18^ ^)^ After quality filtering, data were analyzed for carriage of at least one rare (either novel or population‐based minor allele frequency [MAF] <0.005) using the Genome Aggregation Database (gnomAD) version 2.1.1,^(^ ^19^ ^)^ coding, nonsynonymous single nucleotide variant (SNV), or indel in a highly conserved region (Genomic Evolutionary Rate Profiling [GERP] score >1.5). Data were then filtered based on functional prediction of SNVs using Polymorphism Phenotyping version 2 (Polyphen‐2),^(^ ^20^ ^)^ Sorting Intolerant from Tolerant (SIFT) 4G,^(^ ^21^ ^)^ PMut,^(^ ^22^ ^)^ and Mutation Taster.^(^ ^23^ ^)^

HBM pedigrees were analyzed for variants segregating appropriately for autosomal dominant inheritance (i.e., carried by affected individuals, not carried by unaffected individuals); compound heterozygous (i.e., two different mutation within the same gene) and homozygous inheritances were also considered. Replication was sought by scrutinizing similarly filtered data from all UK HBM and AOGC extreme HBM individuals (seeking carriage of the same variant or other variant within the same gene). For this analysis, a threshold TH or lumbar spine (LS) *Z*‐score ≥ +2.5 was used to select the most extreme high‐BMD individuals from both cohorts. Data from 493 AOGC LBM individuals (with threshold LS *Z*‐scores of <0.5 and TH *Z*‐score <1.5) were similarly scrutinized as negative controls. Variants of interest were validated using Sanger sequencing.

### Measurement of intact and C‐terminal FGF23 concentrations

Human intact (iFGF23) and C‐terminal (cFGF23) forms of FGF23 were analyzed by the Immutopics second‐generation Enzyme‐Linked Immunosorbent Assay (ELISA) kits (Immutopics, San Clemente, CA, USA). The iFGF23 assay employs a murine monoclonal antibody and an affinity purified goat polyclonal antibody to detect epitopes within the amino terminal and carboxyl‐terminal portions of FGF‐23, whereas the antibodies in the cFGF23 assay bind to both the intact molecule and large carboxyl terminal fragments of human FGF‐23. Each sample was analyzed in duplicate as per manufacturer's instruction, with interassay coefficient of variation (CV%) <10% across the assay range of 1.5–2200 pg/mL for iFGF23 and 1.5–1400 RU/mL for cFGF23.

### Protein structural modeling

Using the crystal structure of *T. guttata* GalNAc‐T3 in complex with uridine diphosphate (UDP), manganese, and FGF23 (PDB ID: 6S22)^(^ ^24^ ^)^ as the template (80% identity to human GalNAc‐T3), a homology model was built with SWISS‐MODEL.^(^ ^25^ ^)^ Point mutations were introduced and visualized in silico using PyMOL version 2.4 (www.pymol.org/2/). This highly homologous structure, solved at <2 Å resolution with well‐resolved binding of the Mn^2+^ cofactor and the UDP and FGF23 substrates, allows for confident prediction of the functionally relevant structure of human GalNAc‐T3, for which no crystal structure is available.

### Literature review and mapping of all reported *GALNT3* mutations

PubMed (www.pubmed.ncbi.nlm.nih.gov) was searched using the terms “GALNT3,” “GalNac‐T3,” “familial tumoral calcinosis,” “FTC,” “hyperostosis‐hyperphosphataemia syndrome,” and “hyperostosis‐hyperphosphatemia syndrome.” Abstracts were reviewed to identify case reports of individuals with *GALNT3* variants. Clinical details (including BMD) were reviewed; identified *GALNT3* variants were collated.

### Identification of common *GALNT3* variants associated with BMD

GWASs assessing LS and femoral neck (FN) (UK10K BMD^(^ ^26^ ^)^ and GEFOS LS‐ FN‐ BMD^(^ ^8^ ^)^), total body (TB) (life‐course TB BMD GWAS^(^ ^8^ ^)^), and forearm BMD (UK10K BMD) and BMD estimated from heel ultrasound (eBMD) (UK Biobank eBMD^(^ ^27^, ^28^ ^)^) were interrogated for associations within ±50 kb of the coding sequence of *GALNT3*, *FGF23*, *FGFR1*, and *klotho* using the Musculoskeletal Knowledge Portal (MSKKP; https://msk.hugeamp.org), a data‐mining platform that includes genomic data relating to 291 musculoskeletal traits from 269 datasets.^(^ ^29^ ^)^ A publicly available GWAS of serum phosphate concentration, conducted in UK Biobank (http://www.nealelab.is/uk-biobank/), was also interrogated for associations with the same four loci.

A PheWAS was conducted using the MSKKP (https://msk.hugeamp.org/) and GWASATLAS (https://atlas.ctglab.nl), of which the latter is an online database of publicly available summary results statistics from 4,756 GWASs from 473 unique studies across 3,302 unique traits and 28 domains.^(^ ^30^ ^)^ Significance for pleiotropic associations used the traditional genome‐wide significance threshold for SNP‐trait PheWAS (*p* < 5 × 10^−8^).

### Mendelian randomization (MR) using osteoblast eQTL data

We performed a two‐sample MR. The exposure (*GALNT3*‐expression quantitative trait loci [eQTLs]) was identified by searching eQTL data from primary human osteoblasts^(^ ^31^ ^)^ to identify SNPs within 0.5 kB of *GALNT3* associated with *GALNT3* expression levels (*cis*‐eQTLs). Other phosphate‐handling genes (*FGF23*, *FGFR1*, and *klotho*) were similarly evaluated. F‐statistics were calculated to evaluate instrument strength. The outcome comprised BMD as assessed in the GWAS datasets described earlier.^(^ ^26^, ^28^, ^32^ ^)^ We also examined possible associations of these eQTLs with serum phosphate concentration based on the UK Biobank Phosphate GWAS (http://www.nealelab.is/uk-biobank/). MR analyses were based on Wald ratios,^(^ ^33^ ^)^ where a single eQTL SNP was identified, or inverse variance weighted (IVW)^(^ ^34^ ^)^ and generalized IVW,^(^ ^35^ ^)^ where multiple SNPs were available.

## Results

### A HBM pedigree with a segregating *GALNT3* c.1657C > T p.Arg553Trp variant

**Fig. 1:**
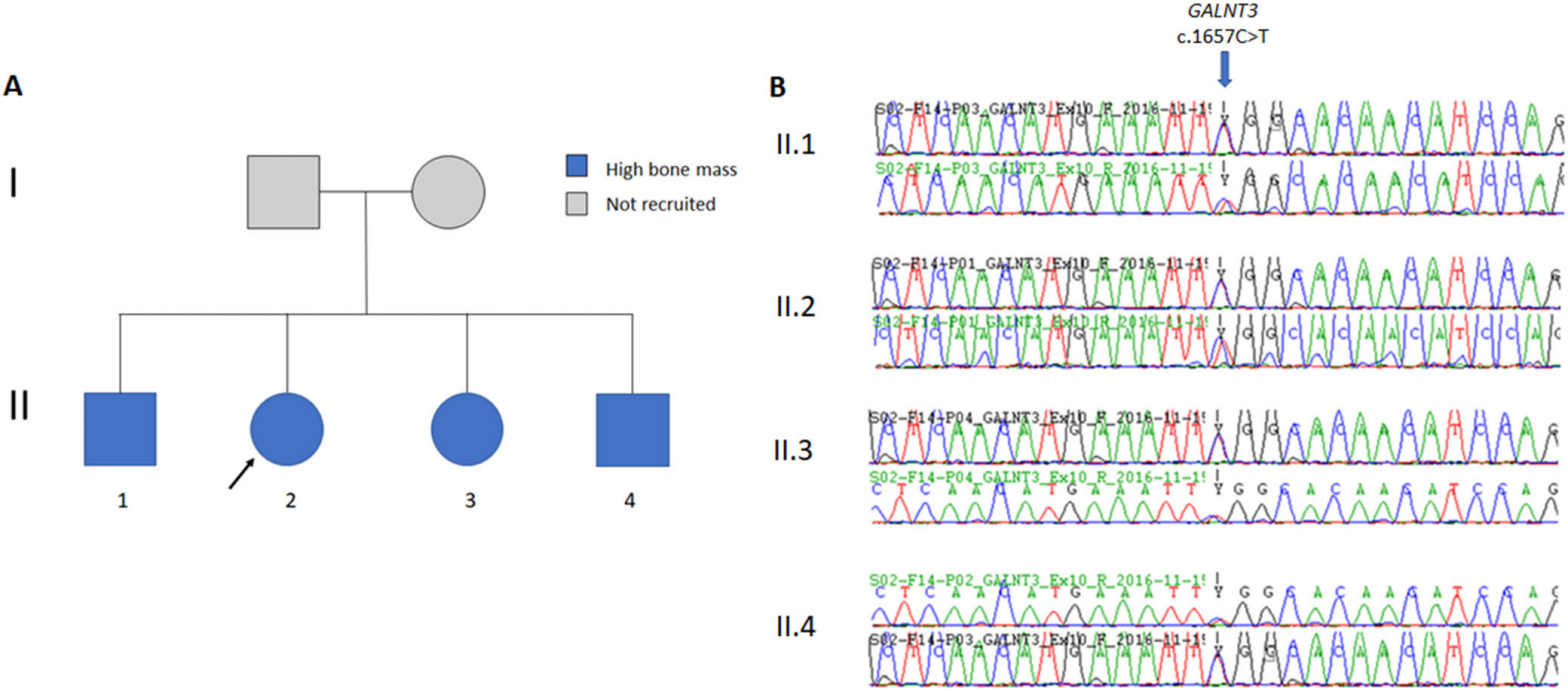
(A) HBM pedigree. (B) Electropherograms with GALNT3 c.1657C > T variant.

A pedigree with unexplained HBM, segregating in an autosomal dominant pattern, was identified within the UK HBM population (Figure 1A). The clinical and biochemical characteristics of each individual are summarized in Table 1, with further details below. All recruited individuals from this pedigree had high BMD *Z‐*scores and high body mass index (BMI). OA and dental overcrowding was common, with all individuals having multiple tooth extractions. There was no history of headaches, nerve compression, or low‐trauma fractures. No individuals had received antiresorptive or anabolic drugs. Neither parent had a history of fracture.

**Table 1:** Clinical and biochemical characteristics of HBM individuals with *GALNT3* c.1657C > T, p.R553W mutations, and a further unrelated HBM individual with a *GALNT3* c.831 *T* > A, p.D277E mutation

	HBM pedigree GALNT3 c.1657C > T, p.Arg553Trp				Additional isolated HBM case GALNT3 c.831 T > A, p.Asp277Glu	
	II.1 brother	II.2 Proband	II.3 sister	II.4 brother	S1	
Age	67	64	59	58	61	
Sex	Male	Female	Female	Male	Female	
Ethnicity	British (white)	British (white)	British (white)	British (whte)	British (white)	
Height (cm)	178	162	160	182	162	
Weight (kg)	86	89	74	94	69	
BMI (kg/m^2^)	27.1	33.9	28.9	28.4	26.3	
DXA L_1_ *Z*‐score	+3.4	+5.2	+3.3	+2.9	+5.2	
DXA TH *Z*‐score	+1.8	+5.1	+2.4	+2.0	+3.2	
Adult fracture	Yes (only digits)	No	No	No	No	
Dental overcrowding	Yes	Yes	Yes	Yes	No	
Torus	Yes	No	No	No	No	
Nerve compression	No	No	No	No	No	
Difficulties with swimming	Not known	No	No	No	Yes	
**Blood tests** ^**a**^						
Adjusted calcium (mmol/L)	–	2.43	–	2.28	–	
Phosphate (mmol/L)	–	1.2	–	0.9	1.3	
iFGF23 (pg/mL)	43.4	52.5	–	22.8	–	
cFGF23 (RU/mL)	25.2	59.5	–	28.0	–	
ALP (IU/L)	50	78	–	70	65	
P1NP (μg/L)	28	27	–	16	15	
CTX (μg/L)	0.05	0.09	–	0.09	0.09	
Osteocalcin (μg/L)	12.2	12.0	–	12.0	6.0	

Abbreviations: ALP, alkaline phosphatase; BMI, body mass index; cFGF23, C‐terminal fragment fibroblast growth factor‐23; CTX, C‐terminal telopeptide of type I collagen; DXA, dual energy X‐ray absorptiometry; iFGF23, intact fibroblast growth factor‐23; P1NP, procollagen type 1 N propeptide.

^a^ Reference ranges: adjusted calcium 2.25–2.70; phosphate 0.8–1.5; iFGF23 28–121; cFGF23 < 100; ALP 20–120; P1NP: postmenopausal female 26–110, mle 20–76; CTX 0.1–0.5; osteocalcin 6.8–32.2.

#### Clinical features

##### Proband

The proband was a 64‐year‐old female with BMD Z‐scores of +5.2 at first lumbar vertebra (L_1_) and + 5.1 at TH. She had no difficulties swimming. She did not have any joint or limb pain and had never fractured. She had required wisdom tooth extractions and was noted to have minimal unilateral hearing loss on a recent hearing test (type of hearing loss not known). She had hypertension (diagnosed at age 34 years) and hypercholesterolaemia (diagnosed at age 64 years). She received menopausal hormone therapy between ages 50 and 57 years and had a hysterectomy and bilateral salpingo‐oophorectomy for stage 1 uterine cancer at age 62 years.

On examination, her height was 1.62 m and weight was 89 kg (BMI 33.9 kg/m^2^). She had mandibular enlargement but no mandibular or maxillary tori and no cranial nerve impingement. She had signs of OA (palpable crepitus in both knees and Heberden's nodes bilaterally) but normal range of movement throughout all joints, including her spine. Radiographs demonstrated degenerative changes in the hands (carpometacarpal joints and several distal interphalangeal joints), knees (medial and lateral compartments), and LS (joint space narrowing and osteophytosis).

##### Brother of proband

The proband's 67‐year‐old brother had BMD *Z* scores +3.4 at L_1_ and + 1.8 at TH. He had no difficulties swimming and denied any joint or limb pain. He had fractured his fingers playing cricket at age 16 years and his thumb playing hockey at age 22 years. He had four teeth removed due to overcrowding. He reported gradual bilateral hearing loss (type not known) over the preceding 10 years. He had hypercholesterolemia and had undergone a prostatectomy for prostate cancer at age 61 years.

On examination, his height was 1.78 m and weight was 86 kg (BMI 27.1 kg/m^2^). He had a small torus arising from the lower left mandible and lower jaw dental overcrowding. He had Heberden's nodes affecting multiple fingers. He had reduced forward flexion in his LS but otherwise normal range of joint movement. Radiographs revealed joint space narrowing and osteophytosis affecting multiple distal interphalangeal joints.

##### Sister of proband

The proband's 59‐year‐old sister resided outside of the UK, so history was obtained over the telephone, physical examination was not performed, and DXA scanning was performed in her home country.

The sister had BMD *Z*‐scores of +3.3 at L_1_ and + 2.4 at TH. She had no swimming difficulties. She had no joint or limb pain and had never fractured. Tooth extractions were performed due to dental overcrowding, and she required an orthodontic brace for 3 years. She had hypertension (diagnosed at age 52 years). She was taking regular calcium/vitamin D supplements. Her height was 1.6 m and weight was 74 kg (BMI 28.9 kg/m^2^).

##### Brother of proband

The proband's 58‐year‐old brother had BMD Z‐scores of +2.9 at L_1_ and + 2.0 at TH. He had no swimming difficulties. He had bilateral ankle pain for over 30 years (i.e., since approximately age 25 years). He had bilateral hip pain and had bilateral hip replacement for OA during the study follow‐up period. Baseline radiographs revealed severe joint space loss at both hips. He had never fractured. He had wisdom teeth removed at age 27 years. He had hypertension (diagnosed at age 50 years), asthma, and depression.

His height was 1.82 m and weight was 94 kg (BMI 28.4 kg/m^2^). He had a broad frame. He had no tori and no cranial nerve impingement. He had bilateral knee crepitus.

#### Sequencing results

A heterozygous missense variant in *GALNT3* (NM_004482:exon 4:c.1657C > T, p.Arg553Trp) was identified, present in all affected siblings (Figure 1). The variant (rs764878326) is rare (GnomAD MAF 0.000016 in European non‐Finnish populations), with a GERP score of 4.47, and predicted to be pathogenic by multiple protein‐prediction algorithms (probably damaging by PolyPhen, damaging by SIFT, and disease‐causing by PMut [pathology score 0.81] and Mutation Taster). No other rare pathogenic variants with appropriate segregation were identified. Sanger sequencing confirmed the variant in each sequenced individual (Figure 1B).

### A further HBM case with a rare heterozygous *GALNT3* variant

Scrutiny of other UK HBM cases, and the HBM AOGC arm, identified an unrelated individual with a different rare heterozygous variant in GALNT3 (c.831 T > A, p.Asp277Glu).

#### Clinical features

##### Proband

This 61‐year‐old female had a BMD *Z*‐score of +5.2 at L_1_ and + 3.2 at TH. She was able to swim but had difficulty floating. She had never fractured. She had OA and fibromyalgia and reported widespread pain in her joints and back with muscle spasms. She reported multiple teeth “breaking” when younger, and she required multiple fillings throughout her 20s and 30s. She had a total hysterectomy at age 40 years and a cholecystectomy at age 60 years.

On examination, her height was 1.62 m and weight was 69 kg (BMI 26.3 kg/m^2^). She had no tori. Cranial nerve examination was normal. She had osteoarthritic changes in her hands and knees. She had reduced range of movement in her cervical spine, but range of movement was normal elsewhere. Radiographs revealed degenerative changes within the hands (joint space narrowing, osteophytosis, and subchondral cysts) and knees (joint space narrowing and osteophytosis).

Her mother had a fragility fracture of her hip. Other family information was limited.

#### Sequencing results

A rare, heterozygous missense variant in *GALNT3* (NM_004482.4:c.831 T > A, p.Asp277Glu) was identified in the proband. This variant (rs139397826) is rare (MAF 0.000025), with a GERP score of 2.59, and is predicted to be pathogenic by multiple protein‐prediction algorithms (probably damaging by PolyPhen, damaging by SIFT, and disease‐causing by PMut [pathology score 0.92] and Mutation Taster).

### LBM controls

#### GALNT3 variants in the LBM cohort

Neither variant (c.1657C > T p.Arg553Trp or c.831 T > A p.Asp.277Glu) was observed on interrogation of the AOGC LBM cohort. No other rare pathogenic *GALNT3* variants were identified in this cohort when the same filtering parameters were used.

### Protein structural modeling predicts that p.Arg553Trp and p.Asp277Glu disrupt GALNT3 function

*GALNT3* encodes the 633 amino acid Golgi‐associated glycosyltransferase, polypeptide N‐acetylgalactosaminyltransferase‐3 (GALNT3). GALNT3 belongs to a large family of Golgi‐associated glycosyltransferases (GalNAc‐Ts) that initiate mucin‐type O‐glycosylation, catalyzing the transfer of N‐acetyl‐D‐galactosamine (GalNAc) from uridine‐diphosphate‐GalNAc (UDP‐GalNAc) to a serine or threonine residue on protein substrates, with relatively broad acceptor substrate specificity.^(^ ^24^ ^)^ GALNT3 consists of a short N‐terminal cytoplasmic tail, a hydrophobic transmembrane domain, a central catalytic (glycosyltransferase) domain, and a C‐terminal ricin B‐type lectin (carbohydrate binding) domain with a β‐trefoil fold. There are two conserved domains within the glycosyltransferase region: the N‐terminal domain (domain A, also known as the GT1 motif) and the C‐terminal domain (domain B, also known as the Gal/GalNAc‐T motif). The ricin B‐type lectin domain binds to GalNAc and contributes to glycopeptide specificity.

**Fig. 2:**
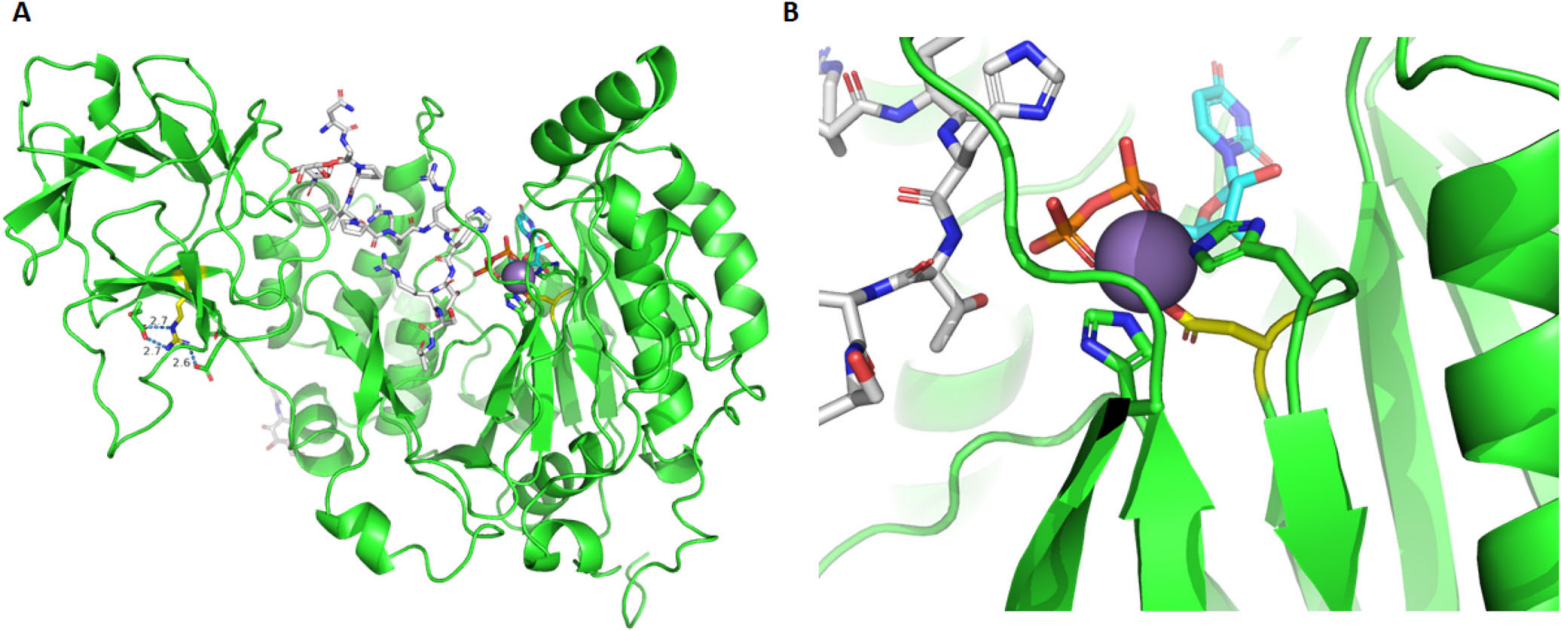
(A) Overview of GALNT3 protein structure with R553 indicated by yellow carbons, with salt‐bridge hydrogen‐bond interactions shown as blue dotted lines. R553W will result in the disruption of these salt‐bridge interactions, causing instability. (B) D277 (Asp277) is shown with yellow carbons and Mn^2+^ in purple, with the other residues coordinating to Mn^2+^ (H279 & H415). The D277E mutation is predicted to affect Mn^2+^ coordination/binding. The UDP substrate, whose binding will depend on Mn^2+^, is shown with cyan carbons and FGF23c with white carbons. Images generated using PyMOL version 2.4.

The c.1657C > T p.Arg553Trp variant in the first pedigree is located within the carbohydrate‐binding lectin domain. Substitution of arginine with tryptophan is predicted to disrupt salt‐bridge interactions, leading to instability of the protein (Figure 2).

The c.831 T > A p.Asp277Glu variant is located within domain A of the glycosyltransferase domain and is part of the D277XH279 motif involved in Mn2+ (manganese) coordination. Mn2+ is key for binding the UDP pyrophosphate group; substitution of aspartate with glutamate is predicted to disrupt Mn2+ coordination and, consequently, UDP binding and, thus, the catalytic function of GALNT3 (Figure 2).

### *GALNT3* variants and bone mass

#### Rare variants

The gnomAD database indicates that the loss‐of‐function (LoF) oe (ratio of observed to expected number of LoF variants) in *GALNT3* is 0.58 (90% CI 0.4–0.86), that is, only 58% of the expected LoF variants are observed. This suggests that *GALNT3* is likely to be under a degree of selection against LoF variants.

Biallelic LoF *GALNT3* variants are known to be associated with the extremely rare autosomal recessive conditions of hyperphosphatemic familial tumoral calcinosis (FTC) and hyperostosis‐hyperphosphatemia syndrome (HHS) (OMIM: 211900). Mice homozygous for a LoF *Galnt3* mutation (Trp589Arg), a model for FTC and HHS, have higher areal BMD compared to wild‐type mice, while heterozygous mice had intermediate BMD.^(^ ^16^ ^)^ Mice homozygous for the mutation also had high cortical bone volume and trabecular number on micro–computed tomography (CT).^(^ ^36^ ^)^

**Fig. 3:**
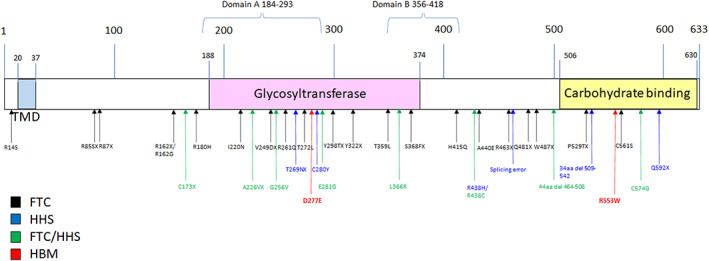
Schematic representation of GALNT3 protein structure. Arrows at sites of known FTC (black) and HHS (blue). Mutations in cases with features of both FTC and HHS are represented in green. HBM mutations are shown in red.

A literature review identified 38 novel/rare LoF *GALNT3* variants in humans, all reported in association with FTC and/or HHS (41 homozygotes, 21 compound heterozygotes) (Table 2). DXA‐measured BMD *Z*‐scores (at TH and/or LS) were reported in only four individuals with *GALNT3*‐associated FTC and HHS (all at age <25 years). Three individuals had normal BMD and one had “extremely reduced” BMD.^(^ ^48^ ^)^ One case series of seven individuals with *GALNT3*‐associated FTC and/or HHS reported that *Z*‐ and *T*‐scores were “within two standard deviations of the mean” for all individuals, apart from one individual who had low *T*‐scores in the context of chronic untreated systemic inflammation.^(^ ^46^ ^)^ *GALNT3* variants associated with FTC/HHS showed no obvious clustering within the structure of the GALNT3 protein or relative to the two variants described in our HBM individuals (Figure 3).

**Table 2:** Summary of published case reports of *GALNT3* variants

Variant	Protein consequence	Clinical consequence	BMD *Z*‐scores	Reference
c.2 T > A	Loss of start codon	HHS	*NR*	Gok et al.^(^ ^37^ ^)^
c.41_58 del	p.Arg14Serfs*8	FTC	*NR*	Garringer et al.^(^ ^38^ ^)^
c.254_255delCT	p.Pro85Argfs*6	FTC	Two females, homozygote, skeletal site not specified. *Z*‐scores of +0.7 (age 10) and 0.0 (age 9)	Kisla et al.^(^ ^39^ ^)^
c.260‐266delGGCAAA	p Arg87Thrfs*19	FTC	Two females (compound heterozygote, ages 6 and 8) –*Z*‐scores reported as “within 2 SD of mean”	Ramnitz et al.
c.484C > T	p.Arg162*	FTC	*NR*	Topaz et al.,^(^ ^40^ ^)^ Ichikawa et al.,^(^ ^41^ ^)^ Demellawy et al.,^(^ ^42^ ^)^ Carmichael et al.^(^ ^43^, ^44^ ^)^
c.485G > A	p.Arg162Gln	FTC	*NR*	Ichikawa et al.^(^ ^45^ ^)^
c.516‐2A > T	p.Cys173Leu fs176*	FTC	Two females (compound heterozygote, ages 6 and 8) –*Z*‐scores reported as “within 2 SD of mean”^(^ ^46^ ^)^	Laleye et al.,^(^ ^47^ ^)^ Ichikawa et al.,^(^ ^41^ ^)^ Ramnitz et al.^(^ ^46^ ^)^
c.516‐2A > G	p.Cys173 fs176*	FTC/HHS	One female (age 16) LS −5.1	Masi et al.^(^ ^48^ ^)^
c.539G > A	p.Arg180His	FTC	*NR*	Sun et al.^(^ ^49^ ^)^
c.659 T > A	p.Ile220Asn	FTC	*NR*	Sun et al.^(^ ^49^ ^)^
c.677delC	p.Ala226Valfs*3	FTC/HHS	*NR*	Ichikawa al.,^(^ ^45^ ^)^ Dauchez et al.^(^ ^50^ ^)^
c.746_749delTCAG	p.Val249Aspfs*8	FTC	One report of one female (compound heterozygote, age 6) with Z‐scores reported as “within 2 SD of mean”	Ramnitz et al.^(^ ^46^ ^)^ Guerra et al.^(^ ^51^ ^)^
c.767G > T	p.Gly256Val	FTC/HHS	*NR*	Rafaelson et al.^(^ ^52^ ^)^
c.782G > A	p.Arg261Gln	FTC	*NR*	Mahjoubi et al.^(^ ^53^ ^)^
c.803‐804insC	p.Thr269Asn fs281*	HHS	*NR*	Ichikawa et al.^(^ ^54^ ^)^
c.815C > A	p.Thr272Lys	FTC	*NR*	Ichikawa et al.^(^ ^55^ ^)^
c.839G > A	p.Cys280Tyr	HHS	*NR*	Gok et al.^(^ ^37^ ^)^
c.842A > G	p.Glu281Gly	FTC/HHS	*NR*	Joseph et al.^(^ ^56^ ^)^
c.892delT*	p.Tyr298Thrfs*5	FTC	One report of one female (compound heterozygote, age 6) with *Z*‐scores reported as “within 2 SD of mean”	Ramnitz et al.^(^ ^46^ ^)^
c.966 T > G	p.Tyr322*	FTC	*NR*	Barbieri et al.^(^ ^57^ ^)^
c.1076C > A	p.Thr359Lys	FTC	*NR*	Ichikawa et al.^(^ ^55^ ^)^
c.1097 T > G	p.Leu366Arg	FTC/HHS	*NR*	Joseph et al.^(^ ^56^ ^)^
c.1102_1103 insT	p.Ser368Phefs*8	FTC	*NR*	Garringer et al.^(^ ^38^ ^)^
c.1245 T > A	p.His415Gln	FTC	*NR*	Yancovitch et al.^(^ ^58^ ^)^
c.1312C > T	p.Arg438Cys	FTC/HHS	One female (compound heterozygote, age 22)^(^ ^59^ ^)^	Yancovitch et al.^(^ ^58^ ^)^ Dumitrescu et al.^(^ ^59^ ^)^ Ramnitz et al.^(^ ^46^ ^)^
			TH +1.9	
			LS +0.3	
			Forearm +1.0	
			One report of one female (age 36) with *T*‐ and *Z*‐scores reported as “within 2 SD of mean”^(^ ^46^ ^)^	
c.1313G > A	p.Arg438His	HHS	*NR*	Olauson et al.^(^ ^60^ ^)^
c.1319C > A	p.Ala440Glu	FTC	*NR*	Finer et al.^(^ ^44^ ^)^
c.1387A > T	p.Arg463*	FTC	*NR*	Campagnoli et al.^(^ ^61^ ^)^
c.1392 + 1G > A	Splicing error	HHS	*NR*	Ichikawa et al.^(^ ^45^ ^)^
c.1441C > T	p.Gln481*	FTC	*NR*	Barbieri et al.^(^ ^57^ ^)^
c.1460G > A	p.Trp487*	FTC	*NR*	Garringer et al.^(^ ^38^ ^)^
c.1524 + 1G > A – skip exon 7	Deletion of 44 amino acids from codons 464–508	FTC/HHS	*NR*	Topaz et al.^(^ ^40^ ^)^ Frishberg et al.,^(^ ^62^ ^)^ Frishberg et al.^(^ ^63^ ^)^
c.1524 + 5G > A – skip exon 7	Deletion of 44 amino acids from codons 464–508	FTC	One report of one female (compound heterozygote, age 36) with *T* and *Z*‐scores reported as “within 2 SD of mean”^(^ ^46^ ^)^	Topaz et al.^(^ ^40^ ^)^ Ramnitz et al.^(^ ^46^ ^)^
c.1584_1585insA	p.Pro529Thrfs*17	FTC	1 male (homozygote, age 29)	Ramnitz et al.^(^ ^46^ ^)^
			No *Z*‐scores but *T*‐score reported as	
			AP spine −3.9	
			TH −3.2. Male sibling (homozygote, age 19 with same variant) reported as having *T*‐ and *Z*‐scores “within 2 SD of mean”	
c.1626 + 1G > A – skip exon 8	Deletion of 34 amino acids from codons 509–542	HHS	*NR*	Ichikawa et al.^(^ ^54^ ^)^
c.1681 T > A	p.Cys561Ser	FTC	*NR*	Dayal et al.^(^ ^64^ ^)^
c.1720 T > G	p.Cys574Gly	FTC/HHS	*NR*	Ichikawa et al.^(^ ^45^ ^)^
c.1774C > T	p.Gln592X	FTC	One female (compound heterozygote, age 22)^(^ ^59^ ^)^	Specktor et al.^(^ ^65^ ^)^ Dumitrescu et al.^(^ ^59^ ^)^ Ramnitz et al.^(^ ^46^ ^)^
			TH +1.9	
			LS +0.3	
			Forearm +1.0	
			One female (compound heterozygote, age 36) with *T*‐ and *Z*‐scores reported as “within 2 SD of mean”^(^ ^46^ ^)^	

*Note*: Reported as per the sequence variant nomenclature guidelines from the Human Genome Variation Society (http://varnomen.hgvs.org/).

Abbreviations: BMD, Bone mineral density; FTC, Familial tumoral calcinosis; HHS, Hyperostosis‐hyperphosphatemia syndrome; LS, Lumbar spine; *NR*, Not reported; SD, standard deviation; TH, Total hip.

**Fig. 4:**
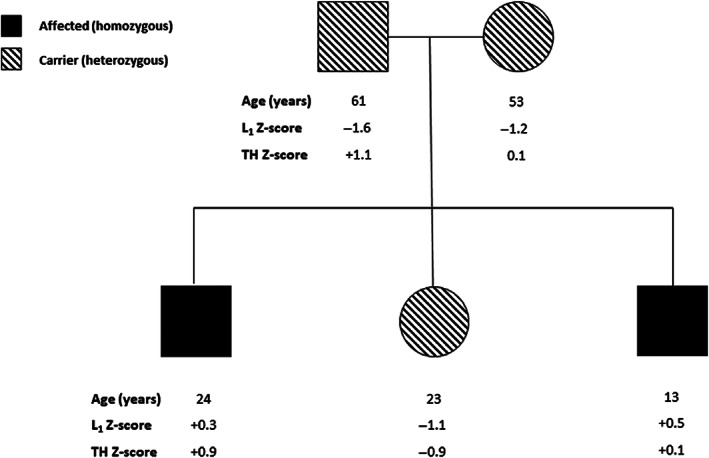
BMD measurements in Italian pedigree with FTC and *GALNT3* c.1524 + 1G > A variant.

We sought to clarify the effect of heterozygous or homozygous *GALNT3* rare LoF variants on bone mass by assessing BMD in a family in whom two individuals with FTC were homozygous for a *GALNT3* variant affecting a splice site (c.1524 + 1G > A) (Figure 4). This variant was previously shown to result in disruption of the intron 7 donor splice site consensus sequence.^(^ ^40^ ^)^ Both homozygous individuals had normal BMD at both LS and TH; in contrast, both carrier parents and their heterozygous sister had Z‐scores <1.0 at LS but normal BMD at TH (Figure 4).

#### Common variants

Common variants at the *GALNT3* locus are associated with BMD, as reported in an earlier GWAS in the AOGC cohort (lead SNP rs1863196 with suggestive association with BMD at TH [*p* = 2.3 × 10^−5^] and LS [*p* = 0.037]).^(^ ^16^ ^)^

Interrogation of subsequent BMD GWASs using MSKKP confirmed the previous suggestive results from AOGC. Significant (*p* < 5 × 10^−8^) associations were observed between SNPs in the *GALNT3* locus (chr2:166,554,098 to 166,701,169) and BMD at TB (lead SNP rs7586085, *p* = 8.64 × 10^−21^),^(^ ^32^ ^)^ femoral neck (lead SNP rs10170839, *p* = 1.2 × 10^−14^),^(^ ^26^ ^)^ and LS (lead SNP rs11680288, *p* = 3.12 × 10^−9^) (Table 3); no significant association was found with forearm BMD,^(^ ^26^ ^)^ or eBMD^(^ ^28^ ^)^ (locus zoom plots for each result: Supplemental Materials Figures S1–S5). Phenome screening revealed an additional genome‐wide significant association with type 2 diabetes (lead SNP rs11686403, *p* = 9.68 × 10^−11^), although not of genome‐wide significance when adjusted for BMI (lead SNP rs6710518, *p* = 2.86 × 10^−5^) (no significant pleiotropic associations were found using GWASATLAS).

**Table 3:** Genome‐wide significant associations of SNPs in the *GALNT3* genomic region (*p* < 5 × 10^−8^) in order of significance

Trait	Lead SNP	MAF	*p* value	*β*	95% CI low	95% CI high	Odds ratio
Total body BMD^(^ ^32^ ^)^	rs7586085	0.41	8.64 × 10^−21^	−0.0532	−0.064	−0.042	
Femoral neck BMD^(^ ^26^ ^)^	rs10170839	0.42	1.2 × 10^−14^	−0.0594	−0.074	−0.045	
Type 2 diabetes^(^ ^66^, ^67^, ^68^, ^69^, ^70^, ^71^, ^72^, ^73^, ^74^, ^75^, ^76^, ^77^, ^78^, ^79^, ^80^, ^81^, ^82^, ^83^, ^84^, ^85^ ^)^	rs11686403	0.41	9.68 × 10^−11^	−0.022	−0.028	−0.017	0.098
Lumbar spine BMD^(^ ^26^ ^)^	rs11680288	0.42	3.12 × 10^−9^	−0.0542	−0.072	−0.037	
Phosphate	rs777356	0.48	2.73 × 10^−5^	−0.01019	−0.015	−0.0054	
eBMD^(^ ^28^ ^)^	rs12692777	0.40	7.20 × 10^−4^	−0.0117	−0.018	−0.005	
Forearm BMD^(^ ^26^ ^)^	rs34100816	0.27	1.78 × 10^−3^	−0.0527	−0.085	−0.02	

*Note*: Lead SNPs for estimated BMD from heel ultrasound (eBMD), forearm BMD, and phosphate are also shown, although these did not reach genome‐wide significance.

Abbreviations: BMD, bone mineral density; CI, confidence interval; eBMD, bone mineral density estimated from heel ultrasound; LDL, low‐density lipoprotein; MAF, minor allele frequency; SNP, single nucleotide polymorphism; *β*, beta value.

Given the effect of GALNT3 on phosphate metabolism via FGF23 (discussed below), we examined whether common variants in genes coding for other members of the FGF23 pathway (*FGF23*, its receptor *FGFR1*, and coreceptor *klotho*) were also associated with BMD. No associations reached genome‐wide significance (Table S1).

### *GALNT3* variants and phosphate homeostasis

#### Rare variants

Biallelic loss‐of‐function variants in *GALNT3* cause disorders of phosphate homeostasis, specifically hyperphosphatemic FTC and HHS,^(^ ^86^ ^)^ acting through the FGF23 pathway.^(^ ^63^, ^87^ ^)^ Although heterozygous carriers of FTC/HHS‐associated *GALNT3* variants do not show any clinical features of FTC or HHS,^(^ ^61^ ^)^ subtle biochemical abnormalities, including slightly elevated phosphate concentrations, have been reported.^(^ ^41^, ^56^ ^)^

Thus, in our HBM cases with heterozygous *GALNT3* variants, we measured phosphate (II.2, II.4, S1), intact FGF23 (iFGF23) (II.1, II.2, II.4), and C‐terminal FGF23 (cFGF23) (II.1, II.2, II.4) concentrations where possible. These results were all in the normal reference range (Table 1).

#### Common variants

Interrogating the UK Biobank GWAS of phosphate concentrations identified strong associations with variants in the *FGF23* locus (lead SNP rs2970818 *p* = 3.23 × 10^−230^) and *klotho* locus (lead SNP rs7324259, *p* = 2.99 × 10^−10^). However, associations with loci containing *GALNT3* and *FGFR1* did not reach genome‐wide significance (Table 3, Table S1).

### Mendelian randomization studies suggest that *GALNT3* expression reduces BMD

We used MR to examine whether *GALNT3*, *FGF23*, *FGFR1*, or *klotho* expression affected BMD. A single *cis‐*SNP, rs13427694, was associated with *GALNT3* mRNA levels in primary human osteoblasts at *p* < 0.05, providing a weak genetic instrument as reflected by an F‐statistic of 7.8 (Table S2). Three *cis‐*SNPs were associated with *FGFR1* mRNA (*F*‐statistic 7.0). One *cis*‐SNP was associated with *klotho* mRNA, which provided an insufficiently strong instrument for further analysis (*F*‐statistic 5.0), whereas no *cis*‐SNP was identified for *FGF23*.

**Fig. 5:**
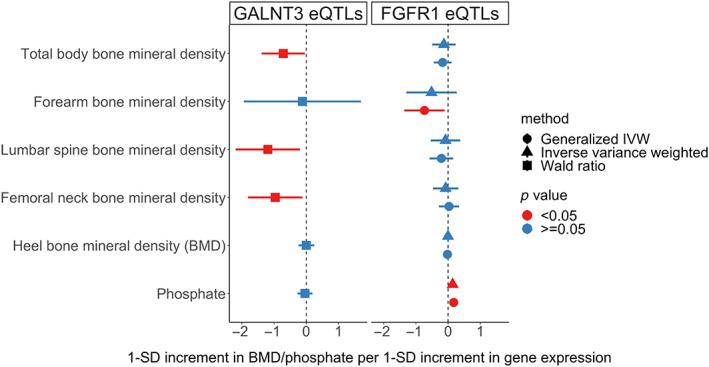
Association of *GALNT3* and *FGFR1* eQTLs with BMD and phosphate.

As shown in Figure 5, subsequent MR analysis suggested that the *GALNT3* osteoblast eQTL was inversely related to FN, LS, and TB BMD, with no associations seen within forearm BMD or heel eBMD, or with serum phosphate levels. While the *FGFR1* eQTL was unrelated to FN, LS, or TB BMD, generalized IVW analysis suggested a decrease in forearm BMD, though IVW estimates crossed the null. Notably, osteoblast *FGFR1* expression was related to an increase in phosphate, particularly in generalized IVW analyses.

## Discussion

We reported two rare heterozygous missense *GALNT3* variants underlying HBM in two independent kindreds. In silico protein modeling predicts that these two variants are likely to result in loss of GALNT3 function, whether through structural instability (c.1657C > T p.Arg553Trp), which may lead to misfolding and more rapid degradation of the protein, or impairment of manganese binding and, consequently, loss of catalytic function (c.831 T > A p.Asp277Glu).

Analysis of common genetic variants from GWASs supports the suggestion from our HBM studies that *GALNT3* plays an important role in regulating bone mass, given loci within this gene show associations at genome‐wide significance with LS, FN, and TB BMD. Though genetic associations do not demonstrate a causal role for a given locus, our MR analysis using *cis*‐eQTL data from primary human osteoblasts suggests that *GALNT3* expression is causally associated with BMD. The eQTL analysis showed that increased expression of GALNT3 was associated with reduced BMD, consistent with our finding of two likely LoF *GALNT3* variants causing an increase in BMD.

Our study demonstrates that the association between *GALNT3* and BMD is supported by both rare and common variants and is consistent with findings from mouse models. As shown previously, drug targets with support from monogenic and population genetic studies are more likely to proceed successfully through the drug development pipeline and be approved.^(^ ^88^, ^89^ ^)^ It is possible that targeting GALNT3‐dependent BMD pathways may open a new avenue in the search for new anabolic treatments for osteoporosis.

It is important to consider potential unwanted effects of targeting GALNT3. Three of the four HBM individuals from the index pedigree had OA; artifactual increases in BMD due to OA were excluded by use of L1 BMD measurements and careful inspection of DXA images. This predisposition to OA is consistent with previous findings from the UK HBM study where individuals with generalized HBM were shown to be at increased risk of developing OA and requiring joint replacement.^(^ ^17^, ^90^ ^)^ “Bone‐forming” features of OA have previously been reported in association with HBM,^(^ ^91^ ^)^ perhaps reflecting an underlying tendency to increased osteoblast activity and excess bone formation, thereby contributing to the osteophytosis observed in several individuals. Dental overcrowding also appeared to be common, but a torus was only seen in one individual. Individuals with these two rare heterozygous *GALNT3* variants appeared to display an otherwise benign clinical phenotype, with an absence of the nerve compression seen with other skeletal dysplasias.^(^ ^5^, ^92^ ^)^

It is known that biallelic loss‐of‐function *GALNT3* mutations cause the rare autosomal recessive disorder FTC and HHS in which deficient GALNT3‐mediated O‐glycosylation results in enhanced cleavage and inactivation of the phosphaturic hormone FGF23, leading to increased/normal inactive C terminal FG23, low intact FGF23, and hyperphosphatemia.^(^ ^87^ ^)^ However, whether these rare variants alter BMD has not been assessed in most cases. Two cases of FTC caused by novel variants in *GALNT3* were reported to have very low BMD.^(^ ^46^, ^48^ ^)^ However, both individuals had other clinical risk factors that could have led to low bone mass. The first individual had hypogonadism and reduced physical activity due to extraskeletal masses,^(^ ^48^ ^)^ while the second individual had untreated chronic systemic inflammation.^(^ ^46^ ^)^ It remains unclear whether these FTC‐associated *GALNT3* variants had any role in directly causing this low BMD alongside disturbing phosphate metabolism.

Acknowledging that we were only able to assess these parameters in a few individuals, the normal phosphate, iFGF23, and cFGF23 concentrations in our heterozygous HBM *GALNT3* cases raised the possibility that *GALNT3* was able to alter BMD independently of the FGF23 pathway. Interrogation of the UK Biobank phosphate GWASs and our MR analysis further supported this hypothesis. In contrast to TB, lumbar, and spinal BMD, our *GALNT3* eQTL was unrelated to circulating phosphate, and though there was a suggestive association between the *GALNT3* genomic region and phosphate, this failed to reach genome‐wide significance.

In contrast to *GALNT3*, no SNPs within the *FGF23* genomic region were associated with any BMD parameter at genome‐wide significance. The same applies to *FGFR1* and *klotho*, the downstream receptor and coreceptor for FGF23, respectively, defects in which are associated with phosphate abnormalities, likely due to loss of FGF23 activity. This further supports the separation of this pathway from effects on BMD. As expected, *FGF23* and *klotho* were strongly associated with phosphate on GWAS, although the association for *FGFR1* did not reach genome‐wide significance in the UK Biobank dataset. *FGFR1 cis*‐eQTLs identified in osteoblast cultures were unrelated to LS, FN, and TB BMD. That said, *FGFR1* expression was associated with reduced forearm BMD in generalized IVW analyses, though no effect was seen in IVW analyses, possibly as the latter incorporated fewer SNPs. Whereas *FGFR1* expression had a smaller effect on BMD compared to *GALNT3* expression, unlike the latter, *FGFR1* expression had a strong effect on serum phosphate levels, providing further evidence of dissociation of GALNT3 and FGF23 function.

Taken together, studies of common genetic variants and gene expression suggest important functional differences between GALNT3 and FGF23/FGFR1/klotho, with common variants in the former predominantly affecting BMD and the latter phosphate homeostasis. Previous studies of GALNT3 glycosylation activity focused on FGF23 as its substrate. Since the effects of GALNT3 on BMD appear to be independent of phosphate homeostasis, these could be mediated by glycosylation of distinct protein(s) primarily involved in regulating bone mass, which are yet to be identified. HBM caused by heterozygous p.Arg553Trp and p.Asp277Glu *GALNT3* variants may result from the disruption of interactions between GALNT3 and these bone regulatory protein(s).

Bone turnover markers (CTX, osteocalcin, and P1NP) in our HBM individuals were either low or toward the lower limit of normal, indicating that bone turnover overall may be suppressed. This is in keeping with previous findings from the UK HBM cohort, where bone turnover markers generally tended to be lower than those in controls.^(^ ^93^ ^)^ This was also previously found to be the case with some individuals with *LRP5* mutations,^(^ ^94^ ^)^ although others reported normal or high turnover.^(^ ^95^, ^96^, ^97^ ^)^ One possible explanation for this is that generalized HBM may be caused by mechanisms that increase osteoblast activity and bone formation when the HBM phenotype is being developed (i.e., during puberty), and these same mechanisms then suppress bone turnover during later life to maintain the increased BMD.^(^ ^93^ ^)^ Another possible explanation is that these HBM variants lead to wnt activation (similar to HBM variants in *LRP5* and *SOST*), causing suppression of bone resorption as a consequence of the known inhibitory action of wnts on osteoclastogenesis. This mechanism underlies the suppression of bone turnover markers that is seen following the use of romosozumab (which leads to wnt pathway activation)^(^ ^4^ ^)^.

The mechanism by which GALNT3 affects BMD without disturbing phosphate homeostasis requires further investigation to avoid precipitating features of FTC or HHS, particularly because there did not appear to be any obvious clustering or segregation of HBM‐associated GALNT3 variants compared to FTC/HHS‐associated GALNT3 variants within the structure of the protein. Furthermore, male mice with W589R‐induced FTC also had infertility, a feature not widely reported in humans with FTC or HHS. GALNT3 is highly expressed in the testis; one case report described a boy with testicular microlithiasis associated with oligozoospermia.^(^ ^61^ ^)^ The finding that common variants in *GALNT3* on GWAS appear to be protective against type 2 diabetes also raises the concern that inactivating GALNT3 may increase the risk of developing this condition.

There are several limitations to our study. Since phenotyping was only performed in a limited number of family members, information about comorbidities was not consistently available in the kindreds used for this study. Phosphate and FGF23 measurements were found to be normal but were only tested at one time point in some of the HBM cases. Serum phosphate concentration is regulated by several hormones, including PTH, and is heavily influenced by food intake, neither of which was quantified or measured. It has been shown that dietary phosphate intake can affect the development of ectopic calcification in FTC mouse models. High dietary intake of phosphate in humans has also been associated with larger FTC lesions. Dietary phosphate intake in studied individuals was not documented, so it is unclear whether this had any effect on the biochemical phenotype observed in these HBM cases. A further limitation is that the eQTL data used in our study were derived from human osteoblasts obtained from a small sample of 95 donors, so the derived genetic instruments were relatively weak, risking weak instrument bias.

In conclusion, we report two rare heterozygous *GALNT3* variants as putative causes of HBM. Our study indicates that c.1657C > T p.Arg553Trp and c.831 T > A p.Asp277Glu variants are likely to cause loss of function GALNT3. We hypothesize that these *GALNT3* variants may affect BMD through mechanisms that are independent of phosphate and FGF23 regulation. This is supported by evidence from population genetic studies where common variants in *GALNT3*, including a GALNT3 cis‐eQTL, are associated with BMD more strongly than with phosphate levels. Further investigation of the mechanism by which this occurs may yield novel anabolic drug targets for osteoporosis treatment.

## Disclosures

The authors have no potential conflicts of interest.

### Peer Review

The peer review history for this article is available at https://publons.com/publon/10.1002/jbmr.4795.

## Supporting information


**Table S1.** Top genome‐wide associations for SNPs in genomic regions of FGF23, FGFR1, and KL with BMD parameters and phosphate. Abbreviations: BMD: bone mineral density; CI: confidence interval; eBMD: estimated BMD from heel ultrasound; *FGF23*: fibroblast growth factor‐23; *FGFR1*: fibroblast growth factor receptor 1; FN: femoral neck; *KL*: klotho; LS: lumbar spine; MAF: minor allele frequency; SNP: single nucleotide polymorphism; *β*: beta coefficient.
**Table S2.** *GALNT3* and *FGFR1* osteoblast‐derived eQTLs used as genetic instruments for the exposure in two‐sample MR. The F‐statistic for each genetic instrument is shown together with the method of MR analysis used. Abbreviations: eQTL, expressive quantitative trait loci; *FGFR1*, fibroblast growth factor receptor 1, GIVW, generalized inverse variance weighted; IVW, inverse variance weighted; MR, Mendelian randomization; SNP, single nucleotide polymorphism; *β*, beta coefficient.
**Figure S1.** Locus zoom plot for genome‐wide significant associations in *GALNT3* locus with total body BMD (generated using Musculoskeletal Knowledge Portal)
**Figure S2.** Locus zoom plot for genome‐wide significant associations in *GALNT3* locus with femoral neck mineral density (generated using Musculoskeletal Knowledge Portal)
**Figure S3.** Locus zoom plot for genome‐wide significant associations in *GALNT3* locus with lumbar spine BMD (generated using Musculoskeletal Knowledge Portal)
**Figure S4.** Locus zoom plot for genome‐wide significant associations in *GALNT3* locus with type 2 diabetes (generated using Musculoskeletal Knowledge Portal)
**Figure S5.** Locus zoom plot for genome‐wide significant associations in *GALNT3* locus with type 2 diabetes, adjusted for BMI (generated using Musculoskeletal Knowledge Portal)

## Data Availability

The data that support the findings of this study are available on request from the corresponding author. The data are not publicly available due to privacy or ethical restrictions.
