# Supplementary material for: Rare and Common Variants in GALNT3 May Affect Bone Mass Independently of Phosphate Metabolism
Source: J Bone Miner Res. 2023 Mar 13;38(5):678–91. doi: 10.1002/jbmr.4795 (PMC10729283; doi:10.1002/jbmr.4795)
Supplement: Supplementary file 1 — Table S1. Top genome‐wide associations for SNPs in genomic regions of FGF23, FGFR1, and KL with BMD parameters and phosphate. Abbreviations: BMD: bone mineral density; CI: confidence interval; eBMD: estimated BMD from heel ultrasound; FGF23: fibroblast growth factor‐23; FGFR1: fibroblast growth factor receptor 1; FN: femoral neck; KL: klotho; LS: lumbar spine; MAF: minor allele frequency; SNP: single nucleotide polymorphism; β: beta coefficient. Table S2. GALNT3 and FGFR1 osteoblast‐derived eQTLs used as genetic instruments for the exposure in two‐sample MR. The F‐statistic for each genetic instrument is shown together with the method of MR analysis used. Abbreviations: eQTL, expressive quantitative trait loci; FGFR1, fibroblast growth factor receptor 1, GIVW, generalized inverse variance weighted; IVW, inverse variance weighted; MR, Mendelian randomization; SNP, single nucleotide polymorphism; β, beta coefficient. Figure S1. Locus zoom plot for genome‐wide significant associations in GALNT3 locus with total body BMD (generated using Musculoskeletal Knowledge Portal) Figure S2. Locus zoom plot for genome‐wide significant associations in GALNT3 locus with femoral neck mineral density (generated using Musculoskeletal Knowledge Portal) Figure S3. Locus zoom plot for genome‐wide significant associations in GALNT3 locus with lumbar spine BMD (generated using Musculoskeletal Knowledge Portal) Figure S4. Locus zoom plot for genome‐wide significant associations in GALNT3 locus with type 2 diabetes (generated using Musculoskeletal Knowledge Portal) Figure S5. Locus zoom plot for genome‐wide significant associations in GALNT3 locus with type 2 diabetes, adjusted for BMI (generated using Musculoskeletal Knowledge Portal) [file JBMR-38-678-s001.docx]

**SUPPLEMENTAL MATERIALS**

Supplemental Tables

Table S1: Top genome-wide associations for SNPs in the genomic regions of FGF23, FGFR1 and KL with BMD parameters and phosphate.

| **Closest gene** | **Trait** | **Dataset** | **Lead SNP** | **MAF** | **p value** | **β** | **95% CI low** | **95% CI high** |
| --- | --- | --- | --- | --- | --- | --- | --- | --- |
| *FGF23* | Total body BMD | Life-course total body BMD GWAS | rs144642925 | 0.02 | 3.79E-03 | 0.06 | 0.019 | 0.098 |
|  | FN BMD | UK10K BMD GWAS | rs144642925 | 0.02 | 9.41E-04 | 0.09 | 0.039 | 0.145 |
|  | LS BMD | UK10K BMD GWAS | rs187226015 | 0.03 | 1.05E-03 | -0.1 | -0.16 | -0.042 |
|  | eBMD | UK Biobank eBMD GWAS | rs12578505 | 0.01 | 5.70E-04 | 0.11 | 0.051 | 0.177 |
|  | Forearm BMD | UK10K BMD GWAS | rs12815443 | 0.31 | 3.34E-04 | 0.06 | 0.026 | 0.088 |
|  | Phosphate* | UK Biobank | rs2970818 | 0.1 | 3.24E-230* | 0.13 | 0.12 | 0.14 |
| *FGFR1* | Total body BMD | Life-course total body BMD GWAS | rs10110231 | 0.01 | 8.41E-03 | 0.23 | 0.06 | 0.408 |
|  | FN BMD | UK10K BMD GWAS | rs148065998 | 0.003 | 1.06E-02 | -0.14 | -0.247 | -0.035 |
|  | LS BMD | UK10K BMD GWAS | rs7829871 | 0.16 | 3.74E-04 | 0.05 | 0.022 | 0.074 |
|  | eBMD | UK Biobank eBMD GWAS | rs533490304 | 0.004 | 9.90E-04 | -0.04 | -0.071 | -0.007 |
|  | Forearm BMD | UK10K BMD GWAS | rs17182141 | 0.03 | 2.64E-04 | 0.13 | 0.061 | 0.198 |
|  | Phosphate | UK Biobank | rs2915665 | 0.001 | 2.45E-04 | -0.05 | -0.078 | -0.024 |
| *KL* | Total body BMD | Life-course total body BMD GWAS | rs7319588 | 0.08 | 3.46E-03 | 0.03 | 0.01 | 0.052 |
|  | FN BMD | UK10K BMD GWAS | rs188195525 | 0.004 | 3.11E-03 | 0.16 | 0.055 | 0.258 |
|  | LS BMD | UK10K BMD GWAS | rs563925 | 0.26 | 3.91E-03 | -0.04 | -0.072 | -0.015 |
|  | eBMD | UK Biobank eBMD GWAS | rs150450013 | 0.003 | 1.00E-03 | 0.031 | 0.006 | 0.055 |
|  | Forearm BMD | UK10K BMD GWAS | rs61943416 | Not reported | 8.87E-03 | 0.091 | 0.024 | 0.157 |
|  | Phosphate* | UK Biobank | rs7324259 | 0.23 | 2.99E-10* | 0.023 | 0.016 | 0.03 |

* denotes those associations which reached genome-wide significance (p-value <5 x 10^-8^)

**Abbreviations:** SNP: single nucleotide polymorphism; MAF: minor allele frequency; β: beta coefficient; CI: confidence interval; *FGF23*: fibroblast growth factor-23, *FGFR1*: fibroblast growth factor receptor 1; *KL*: α-klotho; BMD: bone mineral density; FN: femoral neck; LS: lumbar spine; eBMD: estimated BMD from heel ultrasound

Table S2: GALNT3 and FGFR1 osteoblast-derived eQTLs used as genetic instruments for the exposure in two-sample MR. The F-statistic for each genetic instrument is shown together with the method of MR analysis used.

| **Gene** | **SNP** | **Effect allele** | **Other allele** | **Effect allele frequency** | **β** | **Standard error** | **p value** | **Sample size** | **F-statistic** | **F mean** | **Method** |
| --- | --- | --- | --- | --- | --- | --- | --- | --- | --- | --- | --- |
| *GALNT3* | rs13427694 | C | T | 0.04 | 0.054097 | 0.019202 | 0.00342 | 95 | 7.769967 | 7.769967 | Wald Ratio |
| *FGFR1* | rs150512143 | C | T | 0.02 | 0.10861 | 0.038336 | 0.010161 | 95 | 7.857723 | 6.963382 | GIVW |
| *FGFR1* | rs17182190 | T | G | 0.01 | 0.092819 | 0.03324 | 0.013707 | 95 | 7.633298 | 6.963382 | GIVW |
| *FGFR1* | rs7012413 | C | T | 0.22 | -0.01774 | 0.007555 | 0.020709 | 95 | 5.399125 | 6.963382 | GIVW |
| *FGFR1* | rs150512143 | C | T | 0.02 | 0.10861 | 0.038336 | 0.010161 | 95 | 7.857723 | 7.74551 | IVW |
| *FGFR1* | rs17182190 | T | G | 0.01 | 0.092819 | 0.03324 | 0.013707 | 95 | 7.633298 | 7.74551 | IVW |

**Abbreviations**: eQTL: expressive quantitative trait loci; MR: Mendelian randomization; SNP: single nucleotide polymorphism; β: beta coefficient; *FGFR1*: fibroblast growth factor receptor 1; GIVW: generalised inverse variance weighted; IVW: inverse variance weighted

**Supplemental Figures**

**
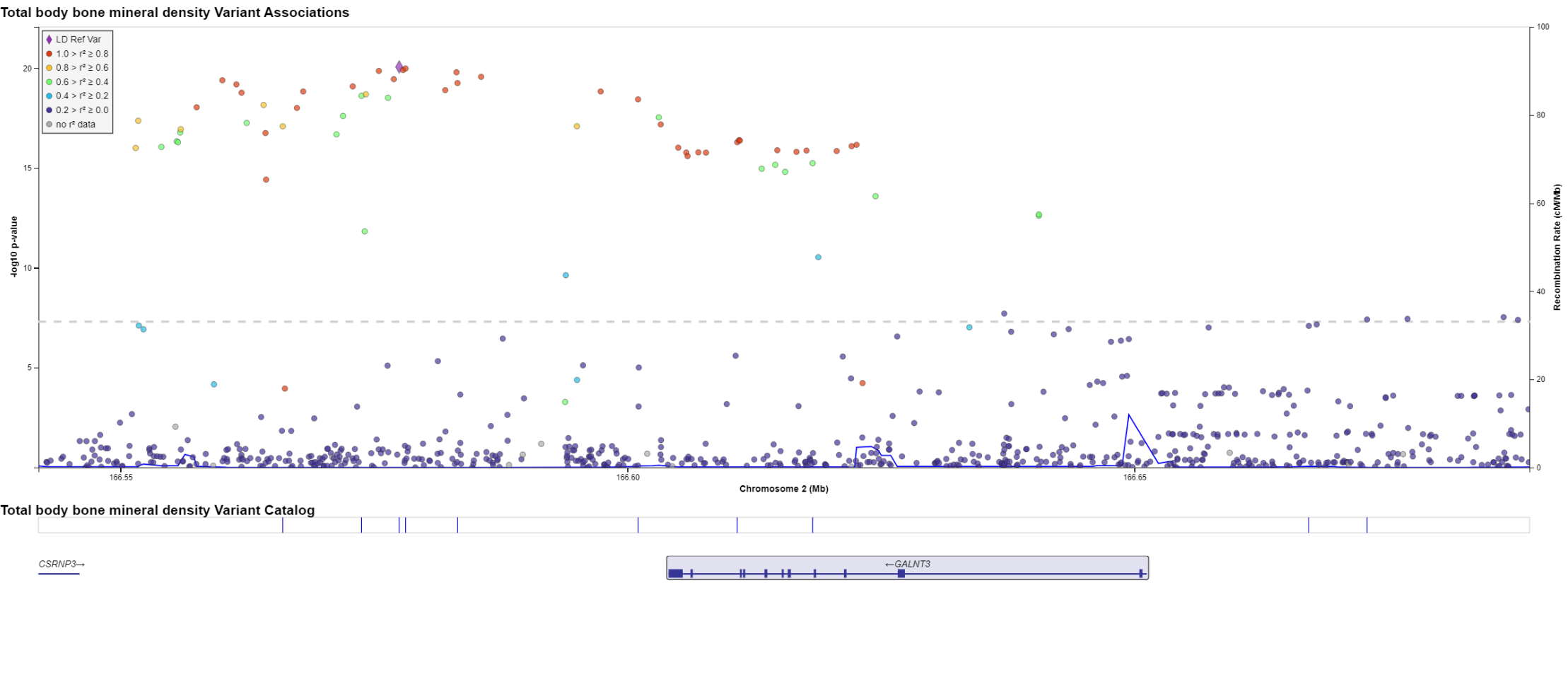
Figure S1 : Locus zoom plot for genome-wide significant associations in the *GALNT3* locus with total body bone mineral density (generated using Musculoskeletal Knowledge Portal)**

**Figure S2: Locus zoom plot for genome-wide significant associations in the *GALNT3* locus with femoral neck mineral density (generated using Musculoskeletal Knowledge Portal)**

**
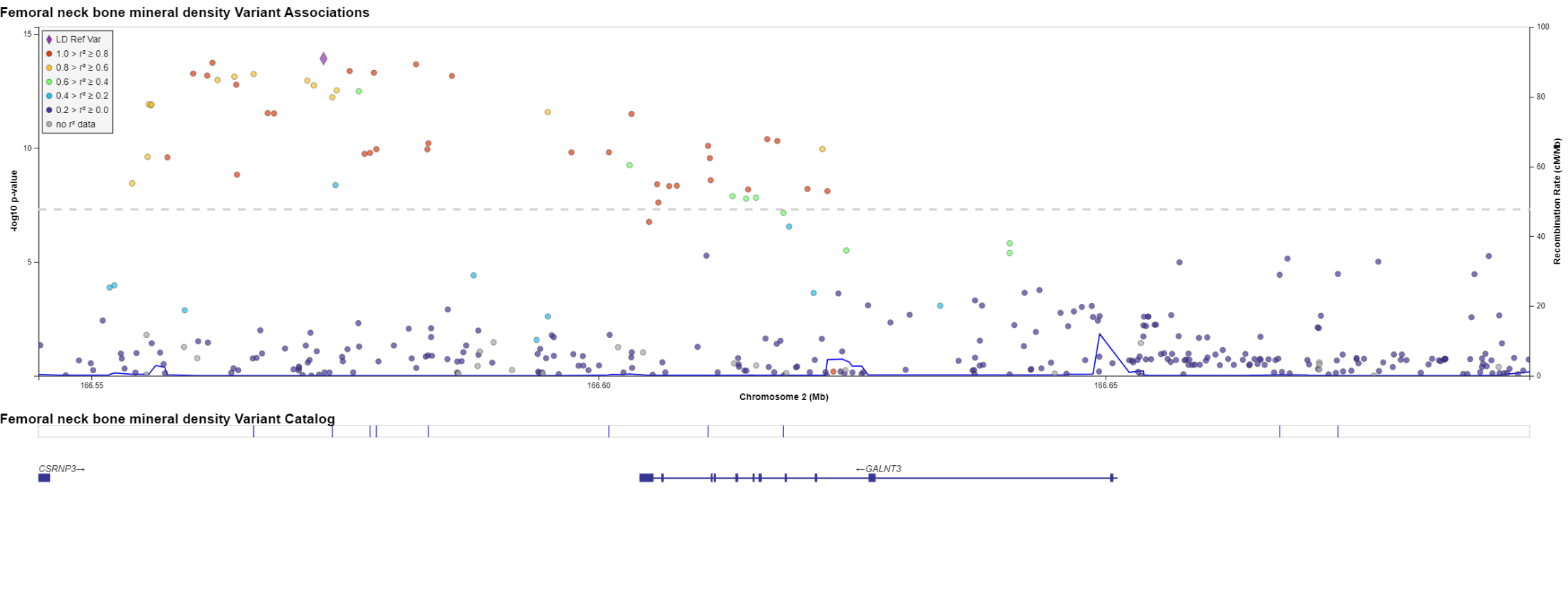
**

**Figure S3: Locus zoom plot for genome-wide significant associations in the *GALNT3* locus with lumbar spine bone mineral density (generated using Musculoskeletal Knowledge Portal)**

**
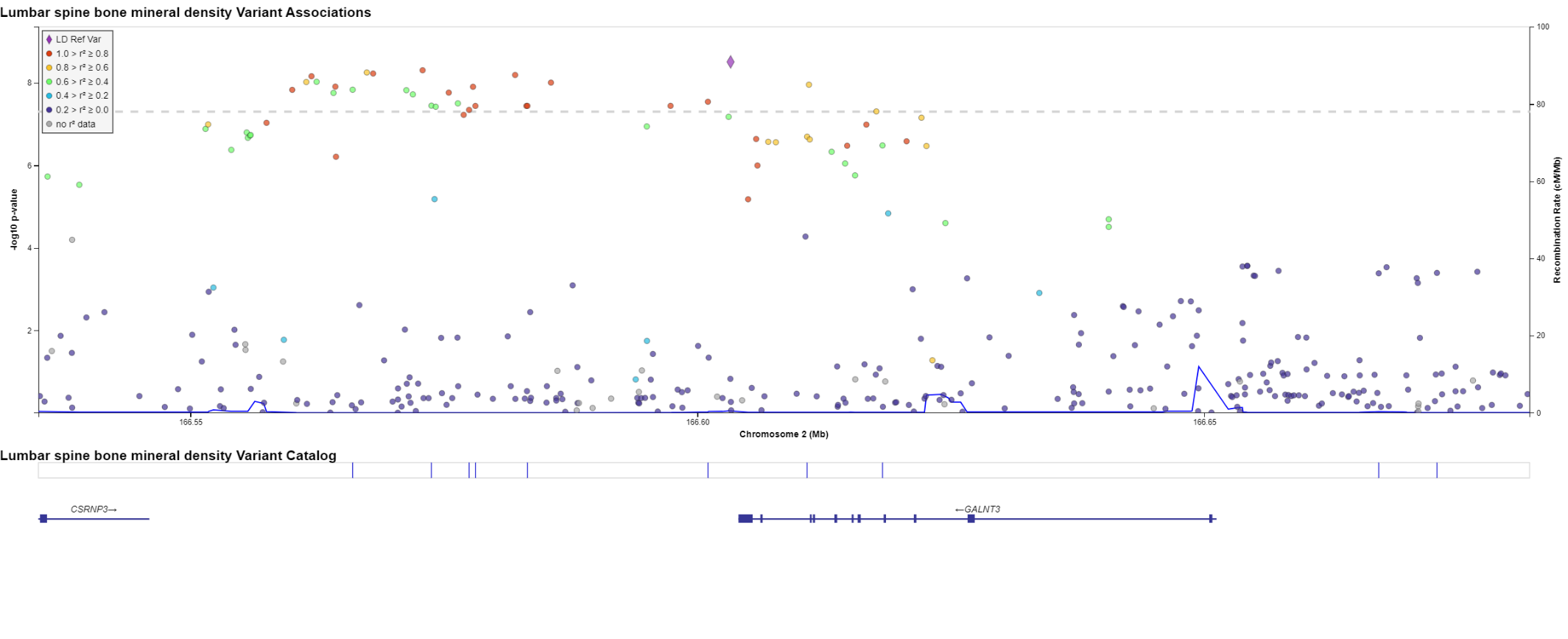
**

**Figure S4: Locus zoom plot for genome-wide significant associations in the *GALNT3* locus with type 2 diabetes (generated using Musculoskeletal Knowledge Portal)**

**
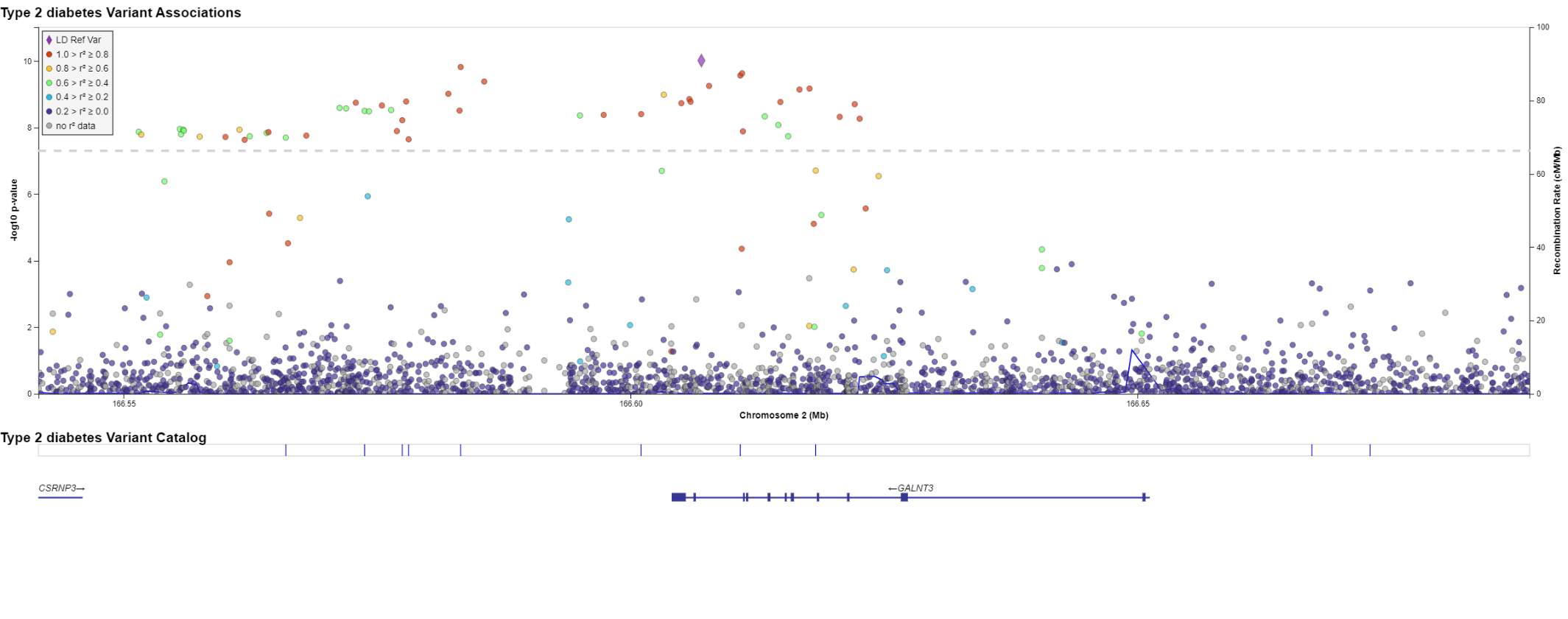
**

**Figure S5: Locus zoom plot for genome-wide significant associations in the *GALNT3* locus with type 2 diabetes, adjusted for body mass index (generated using Musculoskeletal Knowledge Portal)**

**
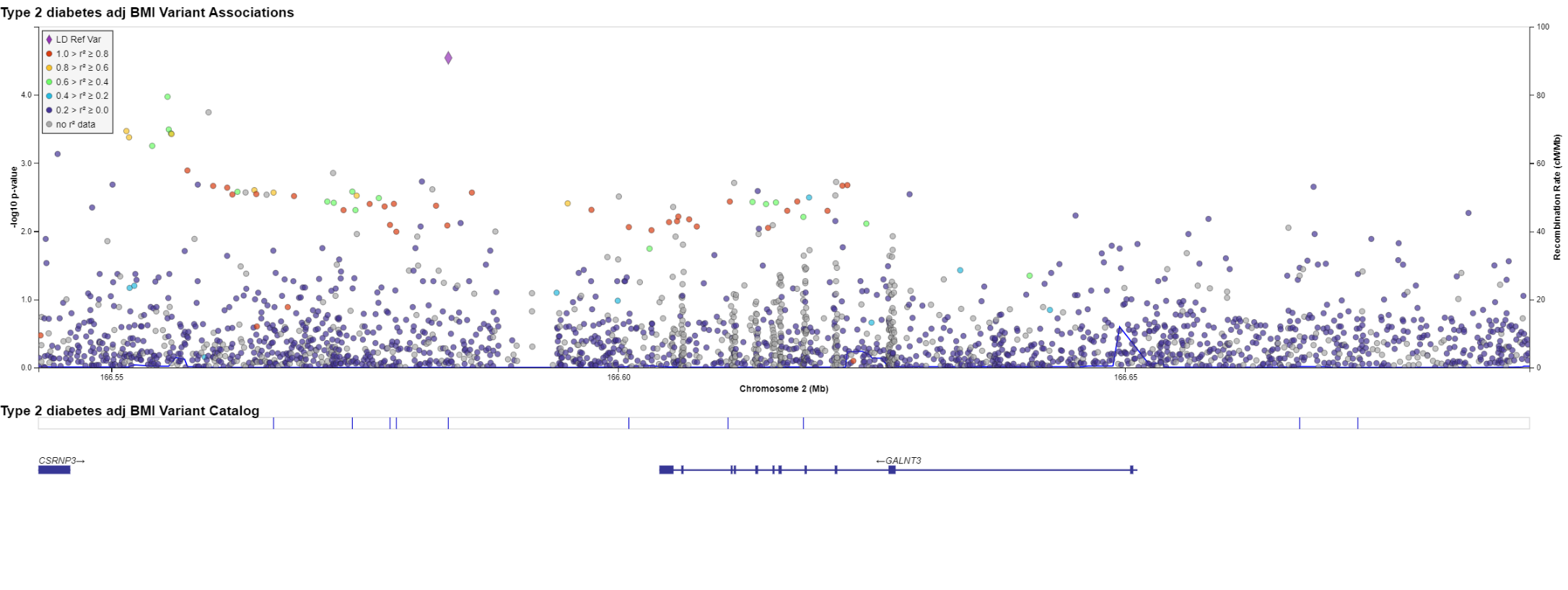
**
